# Redox-Modified Nanostructured Electrochemical Surfaces for Continuous Glucose Monitoring in Complex Biological Fluids

**DOI:** 10.3390/nano14090796

**Published:** 2024-05-03

**Authors:** Sajjad Janfaza, Nandhinee Radha Shanmugam, Pawan Jolly, Prashanthi Kovur, Upasana Singh, Scott Mackay, David Wishart, Donald E. Ingber

**Affiliations:** 1 Wyss Institute for Biologically Inspired Engineering, Harvard University, Boston, MA 02115, USAnandhinee.radhashanmugam@wyss.harvard.edu (N.R.S.); pawan.jolly@wyss.harvard.edu (P.J.); 2 Department of Biological Sciences, University of Alberta, Edmonton, AB T6G 2E8, Canada; kovur@ualberta.ca (P.K.); upasana@ualberta.ca (U.S.); samackay@ualberta.ca (S.M.); dwishart@ualberta.ca (D.W.); 3 Harvard John A. Paulson School of Engineering and Applied Sciences, Cambridge, MA 02139, USA; 4 Department of Pathology, Harvard Medical School, Boston, MA 02115, USA; 5 Vascular Biology Program, Department of Surgery, Boston Children’s Hospital, Boston, MA 02115, USA

**Keywords:** antifouling, glucose biosensor, enzymatic, continuous monitoring, aminoferrocene

## Abstract

Continuous glucose monitoring is valuable for people with diabetes but faces limitations due to enzyme–electrode interactions and biofouling from biological samples that reduce sensor sensitivity and the monitoring performance. We created an enzyme-based electrochemical system with a unique nanocomposite coating that incorporates the redox molecule, aminoferrocene (NH_2_-Fc). This coating enhances stability via electroactivity and reduces nonspecific binding, as demonstrated through cyclic voltammetry. Our approach enables real-time glucose detection via chronoamperometry with a calculated linear range of 0.5 to 20 mM and a 1 mM detection limit. Validated with plasma and saliva, this platform shows promise for robust metabolite detection in clinical and research contexts. This versatile platform can be applied to accurately monitor a wide range of metabolites in various biological matrices, improving patient outcomes.

## 1. Introduction

Diabetes prevalence has drastically increased worldwide, with more than half a billion people affected by this condition [1]. The current prevalence rate of 6.1% places diabetes as one of the leading causes of death globally [1,2], and it is considered a significant contributor to other chronic diseases, including heart attack, stroke, and kidney disease [2,3]. Elevated levels of glucose in the blood, if not recognized early, are extremely life threatening, yet 23% of adults living with diabetes remain undiagnosed in North America [4]. Therefore, the accurate, reliable, and timely monitoring of glucose from human samples (e.g., saliva, plasma, blood, etc.) is essential for the prevention and management of diabetes and its related complications. Although many glucose biosensors based on different transduction principles have been developed, electrochemical-based glucose biosensors utilizing enzymatic sensing currently dominate the market [3,5,6]. These devices harness the inherent electrochemical properties (e.g., reduction or oxidation capabilities) of metabolites, such as glucose, to develop systems that can rapidly quantify the target analyte in complex biological fluids with high reliability. Most glucose biosensors rely on the enzymatic oxidation of glucose mainly due to the high selectivity and stability of glucose oxidase (GluOx) [6,7,8], because this results in better performances in biological fluids and a longer shelf life than those of other technologies. 

Currently, the rapid quantification of glucose levels from finger-prick blood samples is achieved using a glucometer, a handheld biosensing electrochemical device that enables regular monitoring at home. However, several factors, such as deteriorated test strips, reader’s imprecision, improper storage, interference from multiple medications, and environmental conditions, may impact the reliability of these blood glucose measurements [9,10,11,12]. In addition, the sample site may become painful, and wounds from finger-pricks are at risk of infection due to the requirement for repeated testing [3,12]. Also, these devices utilize a single-point measurement mode that is not practical for continuous monitoring, which is required for the optimal detection of hypo/hyperglycemic levels and improved glycemic control in diabetes patients [10,11]. This is important because glucose levels in blood can be influenced by factors of intrinsic nature (biochemical reactions) or exogenous origin (diet or medication). 

Continuous glucose monitors (CGMs) that relay glucose levels in real-time have been developed to overcome these limitations. CGMs can be life changing as they facilitate more personalized insight into glucose level changes during the day, offering patients the power to make more informed treatment decisions and prevent episodes of hypo/hyperglycemia [11,13]. Although many companies have successfully created and commercialized several CGMs, their application for long-term monitoring, to date, remains restricted due to inaccuracy at low glucose concentrations, high costs, the short duration of single implantation, and biocompatibility issues [13,14]. 

One of the key challenges to obtaining accurate and sensitive real-time measurements of glucose concentrations in complex biological fluids results from the non-specific binding of various biomolecules found in the sample matrix, which can interfere with specific signal detection. For example, biosensors exposed to physiological environments for extended periods may exhibit a high background current or signal drift that can compromise the detection sensitivity and analytical accuracy [12]. This is because these sensors are constructed using materials that are prone to passivation by fouling agents (i.e., proteins, amino acids, lipids, etc.) that form an impermeable layer on the electrode surface, affecting charge transfer kinetics between the target analyte and the electrode surface [15]. This effect discourages the application of biosensors for continuous monitoring since fouling degrades the sensor surface, resulting in a low signal-to-noise ratio and reduced sensitivity [12,15]. Therefore, it is critical to develop methods to address biofouling-related problems to continuously monitor metabolites, such as glucose, in real time over extended periods. 

We have previously addressed the crucial challenge of overcoming signal interference due to biofouling by developing a novel nanocomposite coating based on the glutaraldehyde (GA) crosslinking of conductive nanomaterials, such as reduced graphene oxide (prGOx), dissolved with bovine serum albumin (BSA) in PBS [16]. This nanocomposite formulation not only reduces fouling by creating a more hydrophilic surface at the electrode/electrolyte interface, preventing non-specific adsorption, but also offers enhanced electroconductive properties promoting their electrochemical performance in biological fluids. The prGOx in the nanocomposite formulation serves as a conductive material that facilitates electron transfer across the nanocomposite coating to the underlying electrode; it also provides functional groups required for facile surface modification. We successfully demonstrated the potential of this antifouling coating on an electrochemical sensor for single-point measurements of various biomolecules, including IL-6, PCT, CRP, SARS-CoV NS1, GFAP, and NFL in different biological matrices [17,18,19]. In the present study, we demonstrated that a prGOx/BSA/GA nanocomposite coating can be used to construct a glucose biosensor that addresses the electrode fouling challenges observed in enzyme-based continuous monitoring systems.

In label-based electrochemical sensors, detection is usually achieved through the diffusion of redox molecules, such as potassium ferro/ferricyanide, from electrolyte to electrode surfaces [20]. Although these biosensors are robust and reliable, sometimes redox probes can contaminate electrode systems, giving rise to non-specific signals, which could be overcome by functionalizing a stable redox-active functional molecule, such as a ferrocene derivative [21,22]. Specific recognition between the capture molecule and target analyte will impede the charge transfer between the immobilized redox probe and electrode surface, which can be leveraged to analyze the biosensor performance. Several research groups have developed biosensing platforms modified with redox mediators to detect various analytes including glucose [21,22], enzymes, proteins, and nucleic acids [23]. However, these platforms do not address the challenges related to biofouling that must be eliminated to translate these devices toward successful commercialization. 

Here, we explored whether the functionalization of the prGOx/BSA/GA nanocomposite coating with a redox-based molecule that has low redox potential and good electrochemical stability can be used to engineer a multifaceted biosensing platform for the continuous monitoring of glucose in complex biological fluids. Our results describe the successful fabrication of a redox-embedded nanocomposite sensor and its application for glucose detection in two clinically relevant biological matrices: saliva and plasma. To accomplish this, we covalently coupled the redox molecule, aminoferrocene (NH_2_-Fc; a ferrocene derivative with NH_2_ substitution) to prGOx/BSA/GA. NH_2_-Fc undergoes reversible oxidation and reduction reactions (Fc ↔ Fc^+^) and exhibits good electrochemical behavior that favors a reagentless determination of target analytes. In our platform, the NH_2_-Fc efficiently mediates the enzymatic oxidation of glucose via immobilized glucose oxidase and retains the activity of an enzyme-immobilized glucose biosensor. Also, the low redox potential (~0.2 V) of NH_2_-Fc reduces the background signal from other interfering electroactive species present in biological samples. Through rigorous experimental evaluations and comparative analyses, we demonstrate, for the first time, the potential of a NH_2_-Fc functionalized prGOx/BSA/GA nanocomposite-coated electrochemical sensor for the continuous monitoring of glucose with high sensitivity and selectivity.

## 2. Materials and Methods

### 2.1. Nanocomposite Deposition

Screen-printed carbon electrodes (SPCEs) (DropSens, Metrohm, Riverview, FL, USA) were cleaned with ethanol (Sigma Aldrich, Burlington, MA, USA), polished with Kimwipes, and washed with deionized water. The SPCEs were dried, and oxygen plasma-treated at 0.5 mbar and 50% power for 8 min (Zepto Diener Plasma, Diener Electronics, Ebhausen, Germany). An antifouling nanocomposite coating consisting of pentaamine-functionalized reduced graphene oxide (prGOx; Sigma Aldrich, Burlington, MA, USA) and bovine serum albumin (BSA; IgG-Free, Protease-Free; Jackson ImmunoResearch, West Grove, PA, USA) was prepared in 10 mM phosphate-buffered saline (PBS, pH 7.4) as reported in our previous work [16]. Briefly, the prepared solution was subjected to tip sonication with a 1 s ON/OFF pulse at 50% amplitude for 30 min (Q125, QSonica LLC, Newtown, CT, USA), followed by heating at 105 °C for 5 min. The protein-denatured solution was centrifuged (Centrifuge 5418R, Eppendorf, Framingham, MA, USA) at 16.2 rcf for 15 min to remove excess prGOx/BSA aggregates. A mixture of the prGOx/BSA supernatant solution and 70% glutaraldehyde (GA; Sigma Aldrich, Burlington, MA, USA) was prepared in the ratio of 69:1, applied over the plasma-treated SPCEs, and incubated overnight in a humidity chamber. The prGOx/BSA/GA nanocomposite-deposited SPCEs were washed the following day with PBS on a shaker at 500 rpm for 10 min and dried using a slide spinner. 

### 2.2. Fabrication of the Redox-Functionalized Glucose Sensor

The surfaces of the nanocomposite-deposited SPCEs were activated using carbodiimide chemistry, wherein 400 mM of 1-ethyl-3-(3-dimethylaminopropyl)carbodiimide hydrochloride (EDC; Thermo Fisher Scientific, Waltham, MA, USA) and 200 mM of *N*-Hydroxysuccinimide (NHS; Sigma Aldrich, Burlington, MA, USA) dissolved in 50 mM 2-(N-morpholino)ethanesulfonic acid (MES; Thermo Fisher Scientific, Waltham, MA, USA) buffer at pH 6.2 were added to the sensor and incubated for 30 min at room temperature. The SPCEs were then rinsed with MilliQ water and dried before the addition of redox molecules. Aminoferrocene (NH_2_-Fc; Sigma Aldrich, Burlington, MA, USA) dissolved in PBS at a 1 mg/mL concentration was added to the working electrode surface and incubated for 45 min, and then rinsed with PBS via agitation at 500 rpm for 5 min and dried. Following the functionalization of NH_2_-Fc, the working electrodes were selectively spotted with 2 μL of an enzymatic solution obtained by mixing glucose oxidase at 80 mg/mL with GA in a 1:1 *v*/*v* ratio. The glucose oxidase-immobilized SPCEs were dried overnight at 4 °C to let the enzyme composition adhere to the prGOx/BSA/GA nanocomposite-coated surfaces. When the electrode surface was completely dry, it was washed gently with PBS and kept stored at 4 °C until use.

### 2.3. Electrochemical Measurements

All electrochemical measurements were carried out on an Autolab PGSTAT128N potentiostat (Metrohm, Riverview, FL, USA). Electrochemical impedance spectroscopy (EIS) was used to characterize the interfacial properties of the electrode surface modification at each step of the glucose biosensor fabrication. Cyclic voltammetry (CV) was used to electrochemically characterize the NH_2_-Fc-functionalized prGOx/BSA/GA nanocomposite coating and to determine the oxidation/reduction potential of NH_2_-Fc. The potential for chronoamperometry measurements used for glucose quantification was obtained from CV measurements. Calibration curves were built for glucose concentrations between 0 mM and 50 mM based on chronoamperometry.

### 2.4. Sample Collection

In this study, saliva and plasma samples were collected from a volunteer following ethical guidelines and with informed consent. The saliva samples were obtained using passive drool collection, while the plasma samples were collected through venipuncture and processed to obtain the plasma fraction. Both the saliva and plasma samples were stored at −80 °C. Prior to electrochemical analysis, the samples were thawed at room temperature and thoroughly mixed. The samples were tested both in their natural state and spiked with glucose, each undergoing three tests for accuracy and reliability.

## 3. Results

### 3.1. Fabrication of the Nanocomposite Interface with Confined Redox Probes

**Figure 1:**
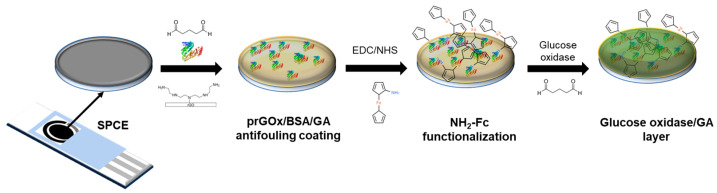
Schematic representing the steps involved in the fabrication of glucose biosensors. The sensor was constructed sequentially with a nanocomposite-based antifouling coating, surface-functionalized redox, and enzyme layers that support glucose detection in biological samples.

The NH_2_-Fc-functionalized glucose biosensor interface was fabricated in three steps (Figure 1) with electrochemical characterization performed after each step (Figure S1). First, the prGOx/BSA/GA nanocomposite coating was drop-cast onto the SPCE surface. The conductive material (prGOx) facilitates electron transfer and improves the electroactive surface area, while the BSA cross-linked with GA provides structural stability and prevents biofouling. Second, the redox mediator, NH_2_-Fc, was covalently functionalized onto the nanocomposite coating through EDC/NHS chemistry. The carbonyl carbon of the NHS ester group undergoes nucleophilic substitution on reaction with the amine group of NH_2_-Fc derivative, resulting in a stable amide bond. Finally, an enzyme layer was coated onto the NH_2_-Fc modified SPCE by drop-casting a solution containing glucose oxidase and GA, and then dried. The GA-crosslinked enzyme layer offers selective permeability for glucose and provides long-term stability for glucose oxidase. 

### 3.2. Electrochemical Characterization of the Redox Immobilized Glucose Sensor

**Figure 2:**
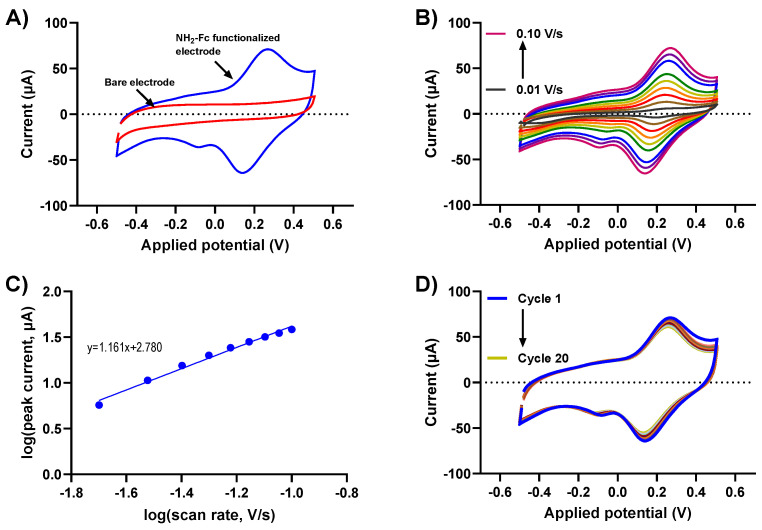
Electrochemical performance of NH_2_-Fc-functionalized prGOx/BSA/GA in PBS. (**A**) Comparison of CV responses for bare and NH_2_-Fc-functionalized nanocomposite-coated SPCEs. (**B**) CV of NH_2_-Fc-functionalized SPCEs measured in PBS at different scan rates. (**C**) Plot demonstrating a linear relationship between logarithm of peak current vs. logarithm of scan rate. (**D**) Cyclic voltammogram recorded in PBS for 20 cycles showing electrochemical stability of NH_2_-Fc-functionalized prGOx/BSA/GA coating.

Understanding the charge transfer kinetics of NH_2_-Fc-functionalized electrode surfaces is crucial to determining their electrochemical performance. Biomolecular binding interactions at the electrode/electrolyte interfaces alter the redox-based charge transfer processes, which can be utilized to design an efficient electrochemical biosensor for various applications. Here, the redox behaviors of the NH_2_-Fc-modified and bare prGOx/BSA/GA nanocomposite-coated SPCEs were obtained using CV measurements in PBS. The CV of NH_2_-Fc-modified SPCEs revealed a defined quasi-reversible oxidation and reduction peak at 0.23 V and 0.15 V, respectively, with a peak separation of 80mV. In contrast, the bare nanocomposite-coated SPCE (Figure 2A) showed no well-defined peaks. This is indicative of the oxidation and reduction of an Fc derivative in NH_2_-Fc during the electrochemical processes, and the obtained peaks were consistent with the redox potential of NH_2_-Fc, confirming the successful functionalization of NH_2_-Fc onto the prGOx/BSA/GA nanocomposite coating. 

The electrochemical oxidation and reduction of NH_2_-Fc functionalization were further evaluated with scan rates ranging from 10 mV/s to 100 mV/s. The voltammograms show a characteristic CV curve with increasing redox peak currents for NH_2_-Fc with an increasing scan rate (Figure 2B). The plot for the log of the scan rate versus the log of the redox peak current exhibited a linear relationship with a slope of 1.1, which is strongly indicative of NH_2_-Fc functionalization contributing toward surface-controlled electrochemical behavior (the expected slope is 1.0) (Figure 2C). 

To further elucidate the stability of NH_2_-Fc functionalization, the prepared prGOx/BSA/GA nanocomposite-coated sensor was subjected to CV measurements in PBS by cycling the potential between −0.5 V and +0.5 V at 100 mV/s for 20 cycles. No significant change in peak current with increasing scan cycles was observed (Figure 2D), confirming the long-term electrochemical stability of the NH_2_-Fc-functionalized prGOx/BSA/GA nanocomposite coating.

### 3.3. Glucose Detection in a Standard Solution

**Figure 3:**
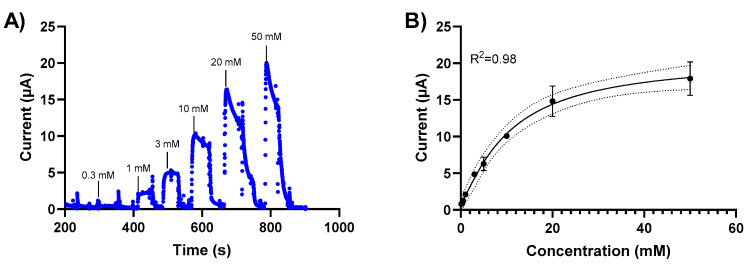
Glucose level-dependent changes in the current response of the NH_2_-Fc-functionalized prGOx/BSA/GA sensor. (**A**) Chronoamperometric response of the NH2-Fc-functionalized prGOx/BSA/GA sensor at 0.2 V in PBS obtained with the successive addition of glucose from 0 to 50 mM. (**B**) Calibration dose–response curve derived from the amperometric signal response. The graph shows a linear response for glucose concentrations between 0 and 20 mM and the saturation for concentrations higher than 20 mM of glucose. Data represented here are from n = 2 replicates.

The sensor response to glucose was measured for glucose concentrations between 0.1 mM and 50 mM using chronoamperometry. Initially, we investigated chronoamperometry measurements at different potentials (0, 0.2 V) to determine the signal sensitivity. We subsequently performed glucose detection measurements using chronoamperometry at 0.2V based on these results. Changes in the current response of the NH_2_-Fc-functionalized prGOx/BSA/GA sensor were measured when exposed to varying glucose concentrations in PBS (Figure 3). A calibration curve was generated by plotting the average of the current response 20 sec after glucose exposure. The sensor exhibits a linear behavior between 0 mM and 20 mM glucose concentrations with a detection limit of 1 mM (Figure 3B) and stable signal response with <10% signal loss over 60 min (Figure S2).

### 3.4. Specificity and Selectivity

**Figure 4:**
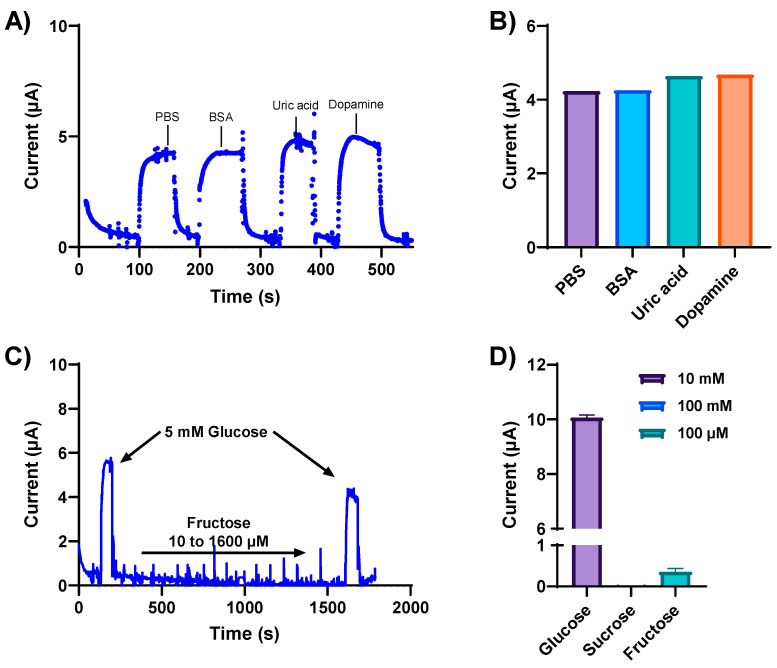
Specificity and selectivity of the glucose sensor were determined in the presence of various interfering electroactive constituents. (**A**) Chronoamperometric response obtained at 0.2 V from the NH_2_-Fc-functionalized glucose biosensor for 5 mM glucose in PBS spiked with the electroactive interferants BSA, uric acid, and dopamine. (**B**) Box plot representation of current response obtained with 2.5 mg/mL BSA, 1 mM uric acid, and 10 μM dopamine spiked separately in a solution containing 5 mM glucose in PBS. (**C**) Amperometry response of the NH_2_-Fc-functionalized glucose sensor with varying concentrations of fructose demonstrating sensor specificity. (**D**) Plot showing the specificity of the developed glucose biosensor for glucose detection compared with other saccharides, sucrose and fructose. Data represented here are from n = 2 replicates.

Biological fluids contain a plethora of metabolites and electrolytes that could potentially interfere with glucose measurements, giving rise to non-specific signal responses. Therefore, the specificity and selectivity of our developed glucose sensor were determined in the presence of various interfering electroactive constituents. The NH_2_-Fc-functionalized glucose sensor was first exposed to physiologically relevant concentrations of potentially interfering substances, such as BSA (2.5 mg/mL), uric acid (1 mM), and dopamine (10 μM), separately spiked in a 5 mM glucose solution. The NH_2_-Fc-functionalized glucose sensor responded selectively toward glucose detection compared to other interferents (Figure 4A). Metabolites such as uric acid and dopamine have positive redox potentials; however, no change in the electrochemical signal response was observed with these potential interferents (Figure 4A,B), and the current response remained almost the same as the test solution spiked with only glucose. This indicates that there is no electrochemical interference from these metabolites, and the electrochemical signal we obtained is from the oxidation/reduction of an Fc derivative NH_2_-Fc immobilized on the antifouling coating.

The specificity of our glucose sensor was further evaluated with varying concentrations of other sugar molecules, such as fructose and sucrose (Figure 4D). The current increased with the addition of 5 mM glucose (Figure 4C), confirming the functional sensitivity of the sensor for glucose detection. Moreover, when subjected to varying fructose concentrations, the same sensor showed no changes in the observed current response, followed by an increase in current response to 5 mM glucose. These results confirm the high specificity of the NH_2_-Fc-functionalized nanocomposite coated sensor for glucose detection.

### 3.5. Glucose Detection in Complex Biological Fluids

**Figure 5:**
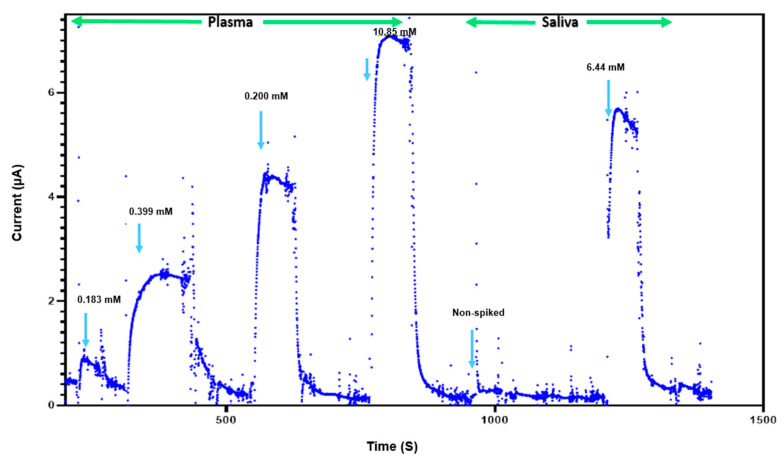
Glucose sensing in complex biological fluids. Response of biosensor to presence and absence of plasma (0.183 mM, 0.399 mM, 0.200 mM, 10.85 mM) and saliva (less than 0.111 mM and 6.44 mM).

The feasibility of glucose detection in true biological samples is essential for determining the usefulness of this sensor for clinical applications. The performance of our NH_2_-Fc-functionalized glucose sensor was investigated using plasma and saliva samples containing different glucose concentrations (Figure 5). The sensor consistently exhibited current responses in the detectable range, indicating that its reliability and robustness are appropriate for potential future clinical use. However, a sensor performance evaluation with real samples will need to be carried out further to evaluate the commercial viability of this glucose sensor. Furthermore, the glucose biosensor shows a linear detection range of 1–10 mM that is well within the expected values for normal blood glucose levels in humans (4–6 mM) compared to other state-of-the-art glucose biosensors reported in prior published studies (Table 1).

## 4. Discussion

Enzyme-catalyzed glucose oxidation has been the standard for developing electrochemical-based glucose biosensors. The general chemical principle involves glucose oxidase, an enzyme that selectively oxidizes glucose to form gluconic acid and hydrogen peroxide (H_2_O_2_) in the presence of oxygen, as indicated by the following reaction:

$$Glucose+H_{2}O+O_{2}\;\overset{Glucose\;oxidase}{\to }Gluconic\;acid+H_{2}O_{2}$$

H_2_O_2_ is further oxidized at the working electrode, leading to the release of two free electrons, constituting an electrochemical signal response proportional to the glucose concentration. However, detection approaches based on O_2_ consumption or H_2_O_2_ production may not be suited for continuous monitoring due to oxygen limitations, especially for sensor systems employed in vivo. An alternative strategy is to replace O_2_ with molecules that can serve as electron mediators, which forms the basis of our work.

The development of an enzymatic electrochemical readout based on a prGOx/BSA/GA nanocomposite-based antifouling coating with a functionalized redox molecule addresses many challenges that need to be overcome to significantly advance metabolomic and diagnostic applications. This new approach reduces cross-reactivity and signal instability in traditional diagnostic devices, which should enable the continuous monitoring of metabolites, such as glucose, over extended periods. 

In this study, the charge transport between the redox center of glucose oxidase and nanocomposite-coated SPCE was achieved using NH_2_-Fc based on the following reaction:

$$Glucose+{NH}_{2}-{Fc}^{+}\;\overset{Glucose\;oxidase}{\to }Gluconic\;acid+{NH}_{2}-Fc+{2H}^{+}$$

The electrochemical responses we measured to establish that NH_2_-Fc mediates the sensing response by tunneling electrons between the electrode and enzyme redox center, which is crucial for the development of glucose biosensors for continuous monitoring. We also showed that the covalent coupling of NH_2_-Fc to the nanocomposite-modified SPCE prevents the leaching of NH_2_-Fc, which is required for building a sensitive detection system. The linear relationship between the glucose concentration and current response demonstrates adequate glucose oxidase loading in the enzyme layer formed via GA crosslinking. Furthermore, the reproducible current response in the presence of various interfering species and the biological sample matrix demonstrates the potential of developing low-cost, rapid, sensitive, and portable electrochemical detection devices for monitoring multiple metabolites, as well as, potentially, for multiplexing these sensors. The simple fabrication methods used and flexibility of modifying the enzyme layer allow for the quick optimization of the developed biosensor platform to different target analytes, opening new avenues for continuous monitoring for various research and clinical applications. In addition, a multivariate analysis can be effectively performed by leveraging advancements in machine learning, resulting in a more accurate and rapid biosensor.

## 5. Conclusions

In summary, we reported the development of electrochemical sensors that address critical challenges in glucose detection and that can potentially offer a new approach for continuous glucose monitoring. The high sensitivity and selectivity of this glucose sensor is based on the use of a nanocomposite-based antifouling coating, a functionalized (Fc derivatized) ferrocene-based redox mediator, and a redox enzyme. The NH_2_-Fc exhibits reversible redox behavior, facilitating electron transport with the redox center of glucose oxidase and supporting the regeneration of enzyme activity for the continuous determination of glucose. A clinical application of the developed platform was demonstrated in plasma and saliva samples, and it showed no interference from other electroactive species. The demonstration that glucose detection can be carried out in complex biological fluids and in the presence of potential interferents expands the application scope of the continuous sensing of glucose as well as other small metabolites. This versatility broadens its potential applications in clinical diagnostics, metabolic profiling, and drug development, in addition to personalized medicine, and, hence, potentially impacts a broad range of healthcare practices.

## Figures and Tables

**Table 1:** Comparison with reported state-of-the-art glucose biosensors.

Platform	Redox	Detection Method	Linear Range	Detection Limit	Comments
Pyrolytic carbon [20]	Potassium ferro/ferricyanide	Amperometry	0.001–1	0.4 µM	Redox species in solution
Gold [24]	GOx modified with ferrocencecarboxaldehyde	Cyclic voltammetry	1.0–5.0 mM	5.2–210 µM	Variable response due to high oxygen levels
Glassy carbon electrode [25]	Poly(glycidyl methacrylateco-vinylferrocene) redox copolymer	Amperometry	0.5 to 6 mM	3 µM	Requires FAD as a cofactor
Multiwalled carbon nanotubes [26]	Osmium redox polymer	Voltammetry /amperometry	-	-	Moderate stability
ZnO Nanorods [27]	-	Cyclic voltammetry	1–13.8 mM	1 mM	Non-enzymatic sensing may have selectivity issues
prGOx/GA/BSA (This work)	NH_2_-Fc	Amperometry	0.0–10 mM	1 mM	Scalable and cost effective

## Data Availability

Data are contained within the article.

## Supplementary Materials

The following are available online at https://www.mdpi.com/article/10.3390/nano14090796/s1, Figure S1: EIS characterization of sensor fabrication. Nyquist plot showing the changes in R_ct_ for each step involved in the glucose sensor fabrication. Z’ and Z” represent the real and imaginary part of measured impedance; Figure S2: Stability of glucose detection over the span of 60 min in 1 mM glucose. Bar plot and the dotted line represents average current response obtained every 10 and over 60 min, respectively; Figure S3: The calibration curve of glucose standard solutions using colorimetric assay. Linear regression analysis: y = 9.1417x with an R^2^ of 0.9967, demonstrating a strong correlation between the variables.
