# Supplementary material for: Redox-Modified Nanostructured Electrochemical Surfaces for Continuous Glucose Monitoring in Complex Biological Fluids
Source: Nanomaterials (Basel). 2024 May 3;14(9):796. doi: 10.3390/nano14090796 (PMC11085330; doi:10.3390/nano14090796)
Supplement: Supplementary file 1 [file nanomaterials-14-00796-s001.zip › nanomaterials-2974549-supplementary.pdf]

nanomaterials

MDPI

Article

Redox-Modified Nanostructured Electrochemical Surfaces for Continuous Glucose Monitoring in Complex Biological Fluids

Sajjad Janfaza [1], Nandhinee Radha Shanmugam [1], Pawan Jolly [1], Prashanthi Kovur [2], Upasana Singh [2], Scott Mackay [2], David Wishart [2] and Donald E. Ingber [1,3,4,5,*]

1 Wyss Institute for Biologically Inspired Engineering, Harvard University, Boston, MA 02115, USA; sjanfaza@prostatecentre.com (S.J.); nandhinee.radhashanmugam@wyss.harvard.edu (N.R.S.); pawan.jolly@wyss.harvard.edu (P.J.)
2 Department of Biological Sciences, University of Alberta, Edmonton, AB T6G 2E8, Canada; kovur@ualberta.ca (P.K.); upasana@ualberta.ca (U.S.); samackay@ualberta.ca (S.M.); dwishart@ualberta.ca (D.W.)
3 Harvard John A. Paulson School of Engineering and Applied Sciences, Cambridge, MA 02139, USA
4 Department of Pathology, Harvard Medical School, Boston, MA 02115, USA
5 Vascular Biology Program, Department of Surgery, Boston Children's Hospital, Boston, MA 02115, USA
* Correspondence: don.ingber@wyss.harvard.edu

Supplementary Data

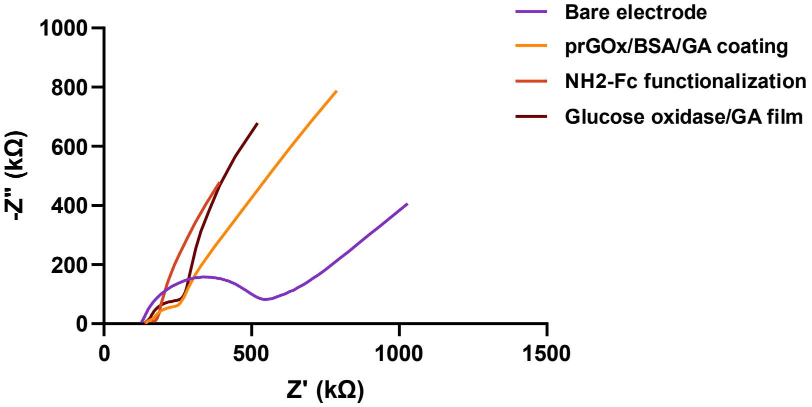

Figure S1. **EIS characterization of sensor fabrication.** Nyquist plot showing the changes in R_{ct} for each step involved in the glucose sensor fabrication. Z′ and Z″ represent the real and imaginary part of measured impedance.

Electrochemical characterization of the developed glucose biosensor was additionaly studied using EIS in 5 mM potassium ferro/ferricyanide in 0.1 mM KCl by applying 10 mV potential from 0.1 Hz to 1 MHz. EIS is highly sensitive to interfacial changes occuring at the electrode/electrolyte surfaces, and hence can provide comprehensive insight to performance and characteristics of the glucose biosensor during the different steps involved in its fabrication. As indicated by the Nyquist plots shown in **Figure S1**, the bare electrode has has a single semicircular region and the smallest charge transfer resistance (R_{ct}). After coating the electrode surface with the nanocomposite , the plot shows two semicircles and an increase in R_{ct} which is indicative of modification of electrode surfaces with prGOx/BSA/GA. However, R_{ct} decreases slightly when NH_2-Fc is covalently coupled to nanocomposite coating, which can be attributed to reduced electroactive behavior of NH_2-Fc in presence of ferro/ferricyanide. Subsequently, the formation of enzyme layer further decreased R_{ct} owing to negatively charged glucose oxidase. These results confirm

successful fabrication of the glucose sensor with the properties described in the main body of this report.

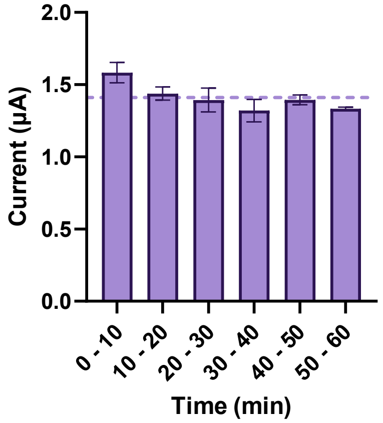

Figure S2. Stability of glucose detection over the span of 60 minutes in 1mM glucose. Bar plot and the dotted line represents average current response obtained every 10 and over 60 minutes, respectively.

The stability of described glucose sensor was evaluated over 60 min in presence of 1 mM glucose concentration. A fresh glucose solution was added every 10 min to account for glucose concentration decay over time as the measurements are conducted under static flow conditions, and averaged amperometric current response over each 10 min window. This approach aimed to demonstrate sensor stability over time, with observed signal loss being <10% which can be attributed to sensor drift.

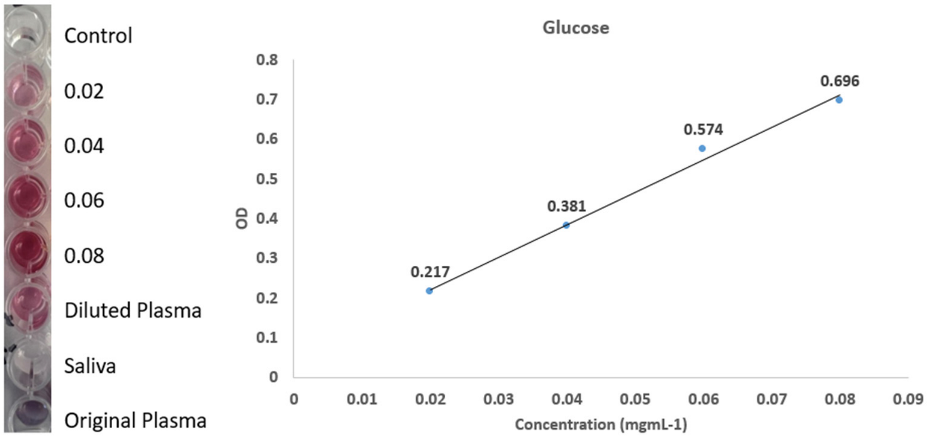

Figure S3. The calibration curve of glucose standard solutions using colorimetric assay. Linear regression analysis: y = 9.1417x with an R^2 of 0.9967, demonstrating a strong correlation between the variables.

A glucose colorimetric assay was performed to determine the glucose concentration in our plasma and saliva samples. A calibration curve was constructed using a series of standard glucose solutions with known concentrations. Each standard solution was subjected to the same colorimetric assay, and the resulting absorbance values were recorded at 540 nm. The absorbance values were then plotted against the known glucose concentrations to create the calibration curve. A linear relationship between the glucose concentration and the absorbance values was observed, as demonstrated by the calibration curve.

By measuring the absorbance of our experimental samples and referencing the curve, we were able to determine their respective glucose concentrations.
